# Neonatal *Candida* infections in Brazil: study on virulence and antifungal susceptibility with predominance of non-*albicans Candida*

**DOI:** 10.1007/s42770-026-01933-9

**Published:** 2026-05-26

**Authors:** Natalia Daiane Garoni Martins, Fabíola Lucini, Bianca Canupa Mancuzo Nascimento, Túlio Máximo Salomé, Nathalie Gaebler Vasconcelos, Letícia Cristina Limiere, Silvane Cavalheiro Silva, Luana Rossato

**Affiliations:** 1 Faculty of Health Sciences, Federal University of Grande Dourados, Rodovia Dourados - Itahum km 12, Cidade Universitária, Dourados, Mato Grosso do Sul 79804970 Brazil; 2 Hospital Universitário da Universidade Federal da Grande Dourados – HU-UFGD, Dourados, Mato Grosso do Sul Brazil

**Keywords:** *Candida* spp., Hemolysin, Neonatal candidemia, Phospholipase

## Abstract

Neonatal *Candida* infections remain a significant cause of morbidity and mortality in neonatal intensive care units (NICUs), especially among preterm and low-birth-weight infants. Given this context, the study investigated *Candida* isolates from neonatal infections in a Brazilian tertiary hospital, focusing on clinical and epidemiological aspects, antifungal susceptibility, and virulence factors. This retrospective observational study was conducted from 2018 to 2024 in a Brazilian NICU. Clinical and demographic data were obtained from medical records of 40 neonates with culture-confirmed *Candida* infections. Antifungal susceptibility testing followed Clinical and Laboratory Standards Institute (CLSI) guidelines. Hemolysin, phospholipase, and biofilm production were evaluated phenotypically. A total of 57 *Candida* isolates were recovered from 40 neonates. The most frequently identified species were *C. parapsilosis* (33.3%), *C. albicans* (22.8%), and *C. tropicalis* (12.3%). Persistent candidemia was observed in 10/40 (25.0%) cases. The overall mortality rate was 45.0%. Notably, four of the fatal cases were associated with strong biofilm-producing strains, and one of these isolates—*C. lusitaniae*—also exhibited high phospholipase activity. Fluconazole and micafungin were the most commonly used antifungal agents. All tested isolates were susceptible to micafungin, while fluconazole showed variable minimum inhibitory concentrations (MICs). Hemolysin and phospholipase activity were detected in most isolates, and approximately 66% were classified as strong biofilm producers. Non-*albicans Candida* species represented the majority of isolates and showed marked virulence attributes and variable antifungal susceptibility. These findings highlight the importance of local surveillance, species-level identification, virulence profiling, and antifungal susceptibility testing to improve clinical outcomes and manage resistance in NICUs.

**Supplementary Information:**

The online version contains supplementary material available at 10.1007/s42770-026-01933-9.

## Introduction

Neonatal candidemia remains a significant cause of morbidity and mortality in neonatal intensive care units (NICUs), especially among preterm and low-birth-weight (LBW) infants [1]. These neonates are particularly vulnerable due to their immunological immaturity, underdeveloped skin and mucosal barriers, as well as hospital-related iatrogenic factors, such as prolonged use of central venous catheters, mechanical ventilation, parenteral nutrition, and administration of broad-spectrum antibiotics [2, 3].

The incidence of *Candida* infections in neonates varies across populations and geographical regions, ranging from 1.4 to 3.6 per 1,000 live births in general populations, but reaching as high as 20% in very low birth weight (VLBW) infants (≤ 1000 g) [2, 4]​. Despite advances in neonatal care and the introduction of antifungal prophylaxis strategies, mortality associated with neonatal candidemia remains alarmingly high, often exceeding 30%, and survivors face increased risks of long-term neurodevelopmental impairments [2, 5].

Epidemiological studies have highlighted a shift in the etiological landscape of neonatal candidiasis, with an increasing prevalence of non-*albicans* species such as *Candida parapsilosis* and *Candida tropicalis* [6, 7]. These species often exhibit intrinsic or acquired antifungal resistance and enhanced virulence factors, including biofilm formation and the secretion of hydrolytic enzymes ​​ [8, 9].

Furthermore, regional differences in species distribution and antifungal susceptibility profiles underscore the need for local epidemiological surveillance to guide empirical therapy and implement effective infection control strategies [10]. In Brazil, studies describing the clinical and microbiological characteristics of neonatal candidemia are limited, especially those involving data from tertiary care centers [11, 12].

Given the scarcity of data, understanding key parameters is vital to enhance diagnostic accuracy, guide antifungal therapy, and reduce neonatal morbidity and mortality. This study addresses this gap by investigating the epidemiological patterns, clinical risk factors, species distribution, antifungal susceptibility, and virulence traits of *Candida* spp. isolated from neonates over a six-year period in a Brazilian NICU.

## Materials and methods

### Study design

This retrospective study analyzed clinical and microbiological data from neonates with *Candida* infections at a tertiary hospital in Mato Grosso do Sul, Brazil, between December 2018 and December 2024. Data were collected from medical records without follow-up, and all information was anonymized. The study was approved by the Ethics Committee of Universidade Federal da Grande Dourados (UFGD) (Project identification code 76088323.2.0000.5160).

### Patient selection

Neonates hospitalized in the NICU for more than 48 h who developed a positive blood culture for *Candida *spp. were included in the study. Additionally, neonates with *Candida* isolated from other clinically relevant specimens, such as urine, tracheal aspirates, peritoneal fluid, and swab samples, were also considered. Detailed information regarding the anatomical sites of isolation is provided in Supplementary Table 1.

Neonatal patients were defined as all infants hospitalized in the NICU or Neonatal Intermediate Care Unit, regardless of chronological or corrected age at the time of diagnosis. This operational definition was adopted based on the institutional care setting and reflects the clinical profile of the population served, which is predominantly composed of preterm and very low birth weight infants who often remain hospitalized beyond the conventional neonatal period.

### Data collection and definitions

Data collected included demographic characteristics, maternal variables, and clinical outcomes. Potential risk factors were also assessed, including prior exposure to broad-spectrum antibiotics, corticosteroids, immunosuppressive agents, and invasive interventions.

Prematurity was defined as birth before 37 completed weeks of gestation, according to the World Health Organization (WHO) criteria. Low birth weight (LBW), very low birth weight (VLBW), and extremely low birth weight (ELBW) were defined as < 2500 g, < 1500 g, and ≤ 1000 g, respectively, in accordance with established international classifications [13]. Infections associated with invasive devices were identified based on their temporal association with catheter use or mechanical support [14, 15]. Persistent infection with *Candida* spp. was defined as the continued recovery of the organism from clinical specimens 72 h or more after the initiation of antifungal therapy. This definition aligns with the IDSA 2016 guidance on serial monitoring until documented clearance [16], and is consistent with the pediatric study [17].

### Species isolation and identification

All positive cultures from patients were included. Yeast isolates recovered from positive cultures were initially identified with the BD Phoenix™ Yeast ID Panel (Becton, Dickinson and Company, Sparks, MD, USA). To confirm species-level identification, isolates were subsequently analyzed by matrix-assisted laser desorption/ionization time-of-flight mass spectrometry (MALDI-TOF MS), using the Microflex™ LT system (Bruker Daltonics, Billerica, MA, USA). For each patient, we analyzed the first isolate, when persistent candidemia occurred, the second isolate was also included. Additional isolates were evaluated only when a different *Candida* species was recovered.

### Antifungal susceptibility testing

Antifungal susceptibility testing was performed on the *Candida* isolates made available by the hospital laboratory, using the broth microdilution method following Clinical and Laboratory Standards Institute (CLSI) guidelines [18], and these same isolates were subsequently used in the additional in vitro assays. Minimum inhibitory concentrations (MICs) were determined for fluconazole (0.125–64 µg/mL), amphotericin B (0.062–8 µg/mL), and micafungin (0.015–8 µg/mL). Standardized fungal suspensions were diluted in RPMI-1640 medium buffered with MOPS and inoculated into 96-well plates at 1–2.5 × 10^3^ CFU/mL, followed by incubation at 37 °C for 24 h. MICs were determined as the lowest concentration yielding complete inhibition of visible growth for amphotericin B, while micafungin MICs were determined based on a 50% reduction in growth, in accordance with CLSI recommendations. For fluconazole, the MIC was defined as the concentration showing a 50% reduction in growth relative to the drug-free control, following CLSI guidelines [18]. *Candida parapsilosis* ATCC 22,019 and *Candida krusei* ATCC 6258 served as quality control strains.

### Virulence assays

#### Hemolysin production

Hemolysin production was assessed using a previously described method, with modifications [19]. Yeast isolates were cultured on Sabouraud Dextrose Agar (SDA) at 37 °C for 24 h, then suspended in Sabouraud Dextrose Broth (SDB) and standardized to 1 × 10^6^ cells/mL. A 5 µL aliquot was spotted in duplicate onto SDA plates supplemented with 3% glucose and 7% defibrinated sheep blood. After drying at room temperature, plates were incubated at 37 °C for 48 h. Hemolytic activity was identified by a translucent halo around the colony, indicating erythrocyte lysis. Colony and halo diameters were measured to calculate the hemolytic index (Hz), defined as the ratio of colony diameter to total diameter. Hemolysin activity was classified as negative (Hz = 1.0), weak (Hz = 0.70–0.99), moderate (Hz = 0.50–0.69), or strong (Hz < 0.50) [20]. Assays were conducted in duplicate. Reference strains of *Candida albicans* (ATCC 90029) served as a positive control strain, while *Candida glabrata* (ATCC 90030) was used as a negative control strain. Although *C. glabrata* can produce hemolysins, it generally does so at lower levels than *C. albicans*, and was included to assess baseline hemolysin production.

#### Phospholipase production

Phospholipase activity was assessed using egg yolk agar, following a previously established protocol with modifications [21]. The medium consisted of SDA supplemented with NaCl, CaCl₂, and 8% sterile egg yolk emulsion. Yeast isolates were cultured in SDB at 37 °C, washed with PBS, and standardized to 1 × 10^6^ cells/mL. A 5 µL aliquot was spotted in duplicate onto the agar and incubated for 48 h at 37 °C. Enzymatic activity was evidenced by a precipitation zone around the colony, due to lecithin hydrolysis. Colony and halo diameters were measured, and the phospholipase index (Pz) was calculated as the ratio of colony diameter to the total diameter (colony + halo). Results were reported as mean Pz values [22]. Assays were conducted in duplicate. Reference strains of *C. albicans* (ATCC 90029) served as a positive control strain, while *C. glabrata* (ATCC 90030) was used as a negative control strain. Despite its potential to produce phospholipase, *C. glabrata* typically expresses these traits at lower levels than *C. albicans*, serving here as a baseline for phospholipase activity.

#### Biofilm production

Biofilm formation was assessed using the crystal violet staining method, adapted from a previously established protocol [23]. Isolates were cultured in RPMI-1640 medium, standardized to 1 × 10^6^ cells/mL, and inoculated into 96-well microtiter plates. After an initial adhesion phase of 90 min, non-adherent cells were removed, and the plates were incubated for 48 h with daily medium replacement. Biofilms were stained with 0.4% crystal violet, washed, and the retained dye was solubilized using 95% ethanol. Absorbance was measured at 595 nm. The assay was performed in duplicate, and results were classified based on OD values: ≤0.131 (no biofilm formation), 0.132–0.262 (weak), 0.263–0.524 (moderate), and > 0.524 (strong) [22, 24, 25]. Reference strains of *C. albicans* (ATCC 90029) served as a positive control strain, while *C. glabrata* (ATCC 90030) was used as a negative control strain. While *C. glabrata* can form biofilms, it typically exhibits lower biofilm-forming ability than *C. albicans*, and was used to assess baseline biofilm formation.

### Statistical analysis

Clinical and demographic variables were analyzed using SPSS Statistics, version 31 (IBM Corp., Armonk, NY, USA). Associations between selected clinical variables and outcomes (mortality and survival) were evaluated using Pearson’s chi-square test and Fisher’s exact test, as appropriate. A *p*-value < 0.05 was considered statistically significant.

## Results

### Clinical and demographic characteristics of neonates with *Candida* spp.

A total of 40 cases were identified during the study period. The majority of neonates were male, 24/40 (60.0%), while 16/40 (40.0%) were female. Thirteen neonates (13/40, 32.5%) were born at ≤ 28 weeks of gestation. Gestational ages between 28 + 1 and 31 + 6 weeks were observed in 10 cases (25.0%), 7 neonates (17.5%) were born between 32 and 36 + 6 weeks, and 10 neonates (25.0%) were born at term (≥ 37 weeks). Among these cases, 18/40 (45.0%) neonates died, and mortality occurred at comparable proportions across the different gestational age categories (Table 1).

Regarding birth weight, 15/40 (37.5%) neonates had ELBW (501–1000 g), 9/40 (22.5%) had VLBW (1001–1500 g), and 16/40 (40.0%) weighed more than 1500 g at birth. Among those who died, 8/18 (44.5%) were classified as ELBW. Mode of delivery was evenly distributed: 21/40 (52.5%) were delivered vaginally, 19/40 (47.5%) by cesarean section (Table 1).

In addition to neonatal characteristics, maternal factors were also evaluated. Lack of prenatal care was identified in 7/40 (17.5%) mothers, while prenatal care was documented in the medical records for 18/40 (45.0%). Information on prenatal care was not available for 15/40 (37.5%) cases. Additional details are provided in Supplementary Table 1. Maternal comorbidities were documented in 18/40 cases (45.0%), and chorioamnionitis was reported in 7/40 (17.5%). Premature rupture of membranes occurred in 14/40 cases (35.0%), and antenatal corticosteroids were administered in 10/40 (25.0%). Regarding parity, 17/40 (42.5%) of mothers were primigravida and 19/40 (47.5%) were multigravida, in 4 cases (10.0%), parity status was not reported. It is noteworthy that maternal comorbidities were present in 10/18 (55.5%) of the neonates who died, and that 9/18 (50.0%) deaths occurred among infants born to multigravida mothers (Table 1).

In terms of clinical conditions and interventions, 12/40 (30.0%) neonates underwent surgical procedures, and 19/40 (47.5%) received postnatal corticosteroids. All neonates 40/40 (100%) had prior exposure to broad-spectrum antibiotics. Parenteral nutrition was administered in 14/40 (35.0%) cases, and in 8/14 (57.1%) of these cases, it was ongoing at the time of the positive *Candida* culture. Central venous catheters were used in 25/40 (62.5%) neonates. Mechanical ventilation was required in 33/40 (82.5%) cases; among these, 4/33 (12.1%) underwent orotracheal intubation and 6/33 (18.2%) received non-invasive support via nasal CPAP. Umbilical venous catheters were placed in 15/40 (37.5%) neonates, while nasogastric or orogastric tubes were used in 21/40 (52.5%) cases (Table 1).

Congenital malformations were identified in 8/40 (20.0%) neonates, predominantly of gastrointestinal origin (6/8, 75.0%). Gastric protectors were administered to 30/40 (75.0%) neonates; in 2 cases (5.0%), this information was not available. These clinical complexities may have contributed to the overall outcomes observed in the study, in which 22/40 (55.0%) neonates survived, while 18/40 (45.0%) died during hospitalization (Supplementary Table 1).

When comparing clinical variables with the outcomes of mortality, no statistically significant differences were observed in the univariable analyses. The distribution of factors such as birth weight, catheter use, mechanical ventilation, and parenteral nutrition was similar between groups, indicating that within the sample size available, none of these variables individually demonstrated a measurable association with the clinical outcomes (Table 1).

**Table 1:** Demographic, maternal, and clinical characteristics of neonates with *Candida* infections by survival outcome

Characteristics	Survival (22)	% (55.0)	Death (18)	% (45.0)	Total	%	Univariable analysis	
							OR (95% CI)	*p*-Value
Sex								
Male	15	68.2	9	50.0	24	60.0	0.46 (0.12–1.69)	0.243
Female	7	31.8	9	50.0	16	40.0	2.14 (0.59–7.76)	0.243
Gestational age								
≤ 28 weeks	7	31.8	6	33.3	13	32.5	1.07 (0.28–4.04)	0.919
28 weeks + 1 day to 31 weeks + 6 days	7	31.8	3	16.7	10	25.0	0.42 (0.09–1.98)	0.464^a^
32 weeks to 36 weeks + 6 days	3	13.6	4	22.2	7	17.5	2.85 (0.45–17.8)	0.381^a^
≥ 37 weeks	5	22.8	5	27.8	10	25.0	1.02 (0.25–4.13)	1.000^a^
Birth weight								
501–1000 g	7	31.8	8	44.5	15	37.5	1.71 (0.47–6.24)	0.412
1001–1500 g	7	31.8	2	11.1	9	22.5		
1501–2000 g	0	0.0	3	16.7	3	7.5	0.26 (0.04–1.49)	0.149^a^
2001–2500 g	1	4.6	0	0.0	1	2.5		
> 2500 g	7	31.8	5	27.8	12	30.0	1.4 (0.39–4.99)	0.604^a^
Delivery method								
Vaginal delivery	11	50.0	10	55.5	21	52.5	1.25 (0.35–4.36)	0.725
Cesarean delivery	11	50.0	8	44.5	19	47.5	0.8 (0.22–2.79)	0.726
Maternal conditions								
No prenatal care	3	13.6	4	22.2	7	17.5	0.55 (0.1–2.87)	0.689^a^
Maternal comorbidities	8	36.3	10	55.5	18	45.0	2.18 (0.61–7.8)	0.225
Chorioamnionitis	3	13.6	4	22.2	7	17.5	1.81 (0.34–9.4)	0.68^a^
Premature rupture of membranes	8	36.3	6	33.3	14	35.0	0.87 (0.23–3.24)	0.842
Antenatal corticosteroid use	6	27.3	4	22.2	10	25.0	0.76 (0.17–3.26)	1.000^a^
Primigravida	10	45.4	7	38.9	17	42.5	0.83 (0.23–2.9)	0.775
Multigravida	10	45.4	9	50.0	19	47.5	1.2 (0.34–4.18)	0,775
Neonatal clinical risk factors and interventions								
Previous surgery	5	22.8	7	38.9	12	30.0	2.16 (0.54–8.56)	0.315^a^
Postnatal corticosteroid use	13	59.0	6	33.3	19	47.5	0.34 (0.95–1.26)	0.105
Parenteral nutrition	6	27.3	8	44.5	14	35.0	2.12 (0.56–7.99)	0.257
Central venous catheter	11	50.0	14	77.8	25	62.5	3.5 (0,87 − 14,05)	0.104
Mechanical ventilation	16	72.7	17	94.4	33	82.5	6.37 (0.68–58.95)	0.105
Orotracheal intubation	4	18.2	0	0.0	4	10.0	0.81 (0.67–0.99)	0.114^a^
Nasal CPAP	5	22.8	4	22.2	9	22.5	0.97 (0.21–4.32)	1.000^a^
Umbilical venous catheter	8	36.3	7	38.9	15	37.5	1.11 (0.3–4.02)	0.87
Nasogastric/orogastric tube	10	45.4	11	61.1	21	52.5	1.44 (0.41–5.06)	0.565
Congenital malformations	3	13.6	5	27.8	8	20.0	2.43 (0.49–12.01)	0.43^a^
Use of gastric protectors	17	77.2	13	72.2	30	75.0	0.76 (0.18–3.21)	0.714
Fungal species identified *								
* C. albicans*	8	32.0	4	22.2	12	27.9	0.5 (0.12–2.04)	0.491^a^
Non - *albicans*	16	64.0	13	72.2	29	67.4	2.0 (0.48–8.19)	0.332
* Candida* spp.	1	4.0	1	5.6	2	4.7	-	-

CPAP = Continuous Positive Airway Pressure; g = grams; n = absolute frequency; % = percentage. This table summarizes the most common comorbidities and clinical conditions observed in neonates diagnosed with culture-positive *Candida* spp. infections during hospitalization. * Percentages in the species section were calculated based on the total number of isolates, since some patients presented with more than one isolate (Supplementary Table 1). ^a^ Fisher’s Exact Test

Other clinical conditions associated with *Candida* infections in neonates included jaundice in 13/40 (32.5%) cases, bronchopulmonary dysplasia in 7/40 (17.5%), hypoglycemia in 6/40 (15.0%), and twin pregnancy in 4/40 (10.0%). Less frequently reported diagnoses included multifactorial anemia, hypoalbuminemia, hyponatremia, apnea, septic shock, and electrolyte imbalances. It is important to note that multiple comorbidities were often present in the same patient, reflecting the complex clinical profiles among the cases analyzed. The mean length of hospital stay until the first positive culture was 36.6 days, ranging from 1 to 149 days. The mean age at diagnosis of *Candida* spp. infection was 47.2 days (range: 5–275 days).

*Candida* spp. were most frequently isolated from blood cultures in 21/40 (52.5%) cases, followed by urine cultures in 10/40 (25.0%) and tracheal secretions in 5/40 (12.5%). Other anatomical sites were reported in 1/40 (2.5%) each, including perineal swab, rectal swab, penile secretion, and peritoneal fluid. In some cases, multiple sites were sampled from the same neonate.

After diagnosis, antifungal therapy was initiated in most cases, with drug selection varying across patients. The most frequently used antifungal agent was fluconazole in 18/40 (45.0%) cases, followed by micafungin in 16/40 (40.0%) and liposomal amphotericin B in 1/40 (2.5%). In 5/40 (12.5%) cases, antifungal therapy was either not administered or not reported. The mean duration of antifungal treatment was 15.7 days (range: 1–62 days), reflecting variability in clinical severity and therapeutic response.

Antifungal regimen modification occurred in 9/40 (22.5%) cases, primarily due to lack of clinical response or persistent *Candida* infection. Among neonates with persistent *Candida* infection, 7/10 (70.0%) required changes to their antifungal regimen.

### *Candida* species and diagnostics

Among the 40 neonates diagnosed with *Candida* spp. infections, 10 (25.0%) presented with persistent infection. A total of 57 *Candida* isolates were recovered from the 40 neonates, with *C. parapsilosis* being the most frequently identified species, accounting for 19 isolates across 10 of the cases (25.0%). *Candida albicans* was detected in 12/40 (30.0%) cases, with a total of 13 isolates, followed by *C. tropicalis* in 7/40 (17.5%) cases, and *C. lusitaniae* (*Clavispora lusitaniae*) in 6/40 (15.0%).

*Candida glabrata* (*Nakaseomyces glabratus*) was recovered on four separate occasions from a single neonate (1/40, 2.5%). Both *Candida melibiosica* (*Helenozyma melibiosica*) and *C. krusei* (*Pichia kudriavzevii*) were detected in 2/40 (5.0%) cases each. Less common species included *C. pelliculosa* (*Wickerhamomyces anomalus*) and *C. pulcherrima* (*Metschnikowia pulcherrima*), each identified in 1/40 (2.5%) case. In 2/40 (5.0%) instances, the isolates were reported simply as *Candida* sp., with no species-level identification.

In total, 57 *Candida* isolates were recovered from the 40 cases, indicating that some patients were colonized or infected with more than one species. Based on isolate counts, *C. parapsilosis* accounted for 19/57 (33.3%) of all isolates, followed by *C. albicans* with 13/57 (22.8%), *C. tropicalis* with 7/57 (12.3%), and *C. lusitaniae* with 6/57 (10.5%). *C. glabrata* was responsible for 4/57 (7.0%), while *C. krusei*, *C. melibiosica*, and *Candida* sp. each comprised 2/57 (3.5%). The least frequent species were *C. pelliculosa* and *C. pulcherrima*, each representing 1/57 (1.8%).

Polymicrobial infections occurred in 2/40 (5.0%) neonates. One case involved a triple infection with *C. parapsilosis*, *C. lusitaniae*, and *C. albicans*, while the other combined *C. albicans*, *C. pulcherrima*, and *C. lusitaniae*.

The number of positive fungal cultures per patient ranged from 1 to 7, with a mean of 1.6, indicating substantial variability in fungal burden among the cases analyzed.

### Antifungal susceptibility profile

Antifungal susceptibility testing was performed on the available *Candida* isolates, revealing diverse MIC profiles across species (Table 2). Due to the retrospective design of the study, antifungal susceptibility testing was limited to isolates that were preserved and viable at the time laboratory analyses were initiated. Susceptibility testing was performed on all retrievable isolates, without the application of selective inclusion criteria.

**Table 2:** Minimum inhibitory concentration (MIC) ranges for antifungal agents against *Candida* isolates recovered from neonates

Candida species (*n*)	Fluconazole (µg/mL)	Amphotericin B (µg/mL)	Micafungin (µg/mL)
*C. parapsilosis* (17)	≤ 0.125–0.5	≤ 0.031–1	0.031–4
*C. albicans* (5)	≤ 0.125–0.5	≤ 0.031–0.25	≤ 0.015–0.125
*C. lusitaniae* (3*)*	0.25–0.5	≤ 0.031–0.25	≤ 0.015–0.25
*C. tropicalis* (2*)*	≤ 0.125–8	0.062–0.5	≤ 0.015
*C. krusei* (2)	0.25–32	0.125–1	0.25–0.5
*C. glabrata* (2)	2	0.062	0.125
*C. melibiosica* (1)	2	0.125	≤ 0.015

MIC = Minimum Inhibitory Concentration. Breakpoints and Epidemiological Cutoff Values (ECVs) [18] were applied as follows:

Fluconazole: susceptible ≤ 2 µg/mL for C. albicans, C. tropicalis, and C. parapsilosis; resistant ≥ 64 µg/mL for C. glabrata; C. krusei intrinsically resistant; C. lusitaniae ECV 1.0 µg/m

Amphotericin B: susceptible ≤ 0.5 µg/mL for C. parapsilosis; ECVs: C. albicans 2.0 µg/mL, C. tropicalis 1.0 µg/mL, C. glabrata 2.0 µg/mL, C. krusei 2.0 µg/mL, C. lusitaniae 2.0 µg/mL

Micafungin: susceptible ≤ 0.25 µg/mL for C. albicans, C. tropicalis, and C. krusei; ≤2 µg/mL for C. parapsilosis; ≤0.06 µg/mL for C. glabrata; C. lusitaniae ECV 0.5 µg/mL

*C. melibiosica* has no established breakpoints or ECVs

Among *C. parapsilosis* isolates Micafungin MICs were more variable, reaching up to 4 µg/mL in some cases, which is consistent with the known reduced susceptibility of this species to echinocandins. All *C. albicans* isolates (*n* = 5) showed low MIC values for micafungin, amphotericin B, and fluconazole. *Candida lusitaniae* also demonstrated low MICs for amphotericin B, and fluconazole MICs were similarly low, ranging from 0.25 to 0.5 µg/mL. For *C. tropicalis*, fluconazole MICs ranged widely from ≤ 0.125 to 8 µg/mL, raising concern for possible resistance in at least one isolate. *Candida krusei* (*n* = 2), intrinsically resistant to fluconazole, showed MICs of 0.25 and 32 µg/mL for this drug. *Candida glabrata* (*n* = 2) showed uniformly low MICs for all tested antifungals, with particularly good activity observed for amphotericin B (0.062 µg/mL), aligning with established susceptibility patterns.

Finally, the single isolate of *C. melibiosica* showed a fluconazole MIC of 2 µg/mL, an amphotericin B MIC of 0.125 µg/mL, and a micafungin MIC of ≤ 0.015 µg/mL, suggesting susceptibility to the polyene and the echinocandin tested, but reduced susceptibility to the azole (Supplementary Table 2).

### Hemolysin production by *Candida* isolates

**Fig. 1:**
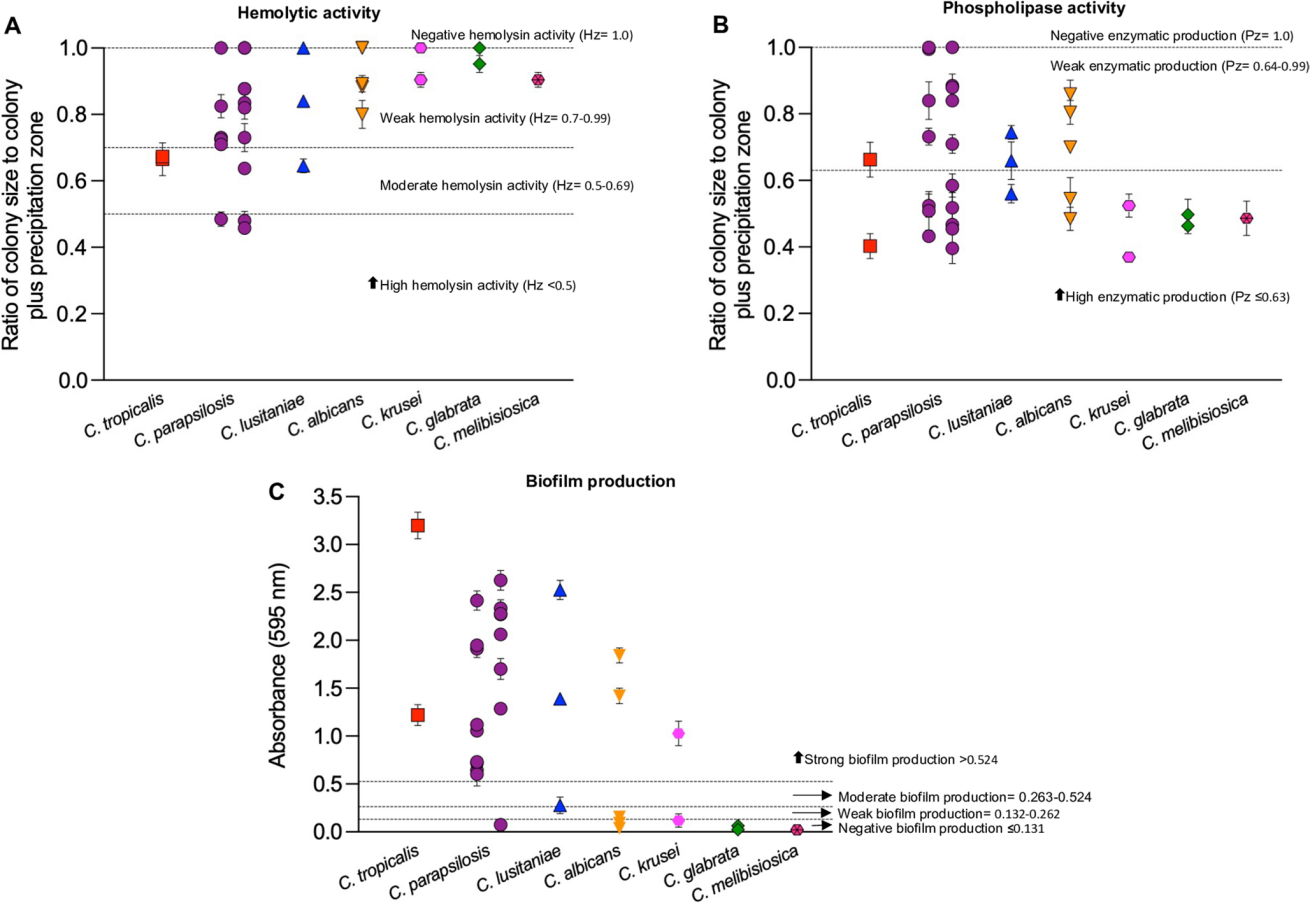
Virulence-related traits of *Candida* isolates recovered from neonates. **A** Hemolytic activity on SDA with sheep blood; Hz index calculated as colony/total diameter (lower values = stronger activity). **B** Phospholipase activity on egg yolk agar; Pz index calculated as colony/total diameter (lower values = stronger activity). **C** Biofilm production measured by OD and classified as none, weak, moderate, or strong. Bars show mean values per species; dotted lines indicate activity thresholds

Hemolysin activity was detected in several *Candida* isolates, with hemolytic index (Hz) values ranging from 0.45 to 1.00. Most isolates of *C. albicans*, *C. glabrata*, *C. krusei*, and *C. melibiosica* demonstrated either weak hemolytic activity (Hz = 0.70–0.99) or none at all (Hz = 1.00). In contrast, *C. lusitaniae* showed one isolate weak activity (Hz = 0.84), one was a non-producer (Hz = 1.00), and one was classified as moderately hemolytic (Hz = 0.64). A similar distribution was observed among the two *C. tropicalis* isolates (Fig. 1A; Table 3).

**Table 3:** Virulence profile of *Candida* isolates

Strains	Hz index	Activity	Pz index	Activity	Biofilm	Activity
*C. tropicalis* 29/22	0.66	Moderate	0.66	Weak	3.2	High
*C. tropicalis* 224/23	0.67	Moderate	0.40	High	1.22	High
*C. parapsilosis* 02/22	0.82	Weak	0.73	Weak	0.64	High
*C. parapsilosis* 03/22	0.63	Moderate	0.88	Weak	1.05	High
*C. parapsilosis* 04/22	0.48	High	0.52	High	1.91	High
*C. parapsilosis* 15/22	0.72	Weak	0.84	Weak	0.72	High
*C. parapsilosis* 18/22	0.73	Weak	0.50	High	0.60	High
*C. parapsilosis* 19/22	0.48	High	0.84	Weak	0.73	High
*C. parapsilosis* 23/22	0.83	Weak	0.71	Weak	1.12	High
*C. parapsilosis* 30/22	0.72	Weak	0.99	Weak	1.95	High
*C. parapsilosis* 112/23	1.0	None	1.0	None	2.41	High
*C. parapsilosis* 197/23	1.0	None	0.88	Weak	1.70	High
*C. parapsilosis* 217/23	1.0	None	0.39	High	0.07	None
*C. parapsilosis* 309/24	0.45	High	0.46	High	1.28	High
*C. parapsilosis* 319/24	0.82	Weak	1.0	None	2.62	High
*C. parapsilosis* 323/24	0.87	Weak	0.51	High	2.33	High
*C. parapsilosis* 327/24	0.73	Weak	0.45	High	2.06	High
*C. parapsilosis* 330/24	0.71	Weak	0.43	High	2.27	High
*C. parapsilosis* 383/24	0.87	Weak	0.58	High	2.27	High
*C. lusitaniae* 16/22	0.84	Weak	0.56	High	2.52	High
*C. lusitaniae* 17/22	0.64	Moderate	0.66	Weak	1.39	High
*C. lusitaniae* 78/23	1.0	None	0.74	Weak	0.27	Moderate
*C. albicans* 384/24	0.88	Weak	0.48	High	0.03	None
*C. albicans* 292/24	0.89	Weak	0.8	Weak	0.15	Weak
*C. albicans* 25/22	1.0	None	0.7	Weak	1.42	High
*C. albicans* 27/22	1.0	None	0.86	Weak	0.09	None
*C. albicans* 28/22	0.8	Weak	0.54	High	1.84	High
*C. krusei* 207/23	0.90	Weak	0.36	High	0.11	None
*C. krusei* 256/23	1.0	None	0.52	High	1.03	High
*C. glabrata* 489/24	0.95	Weak	0.49	High	0.06	None
*C. glabrata* 497/24	1.0	None	0.46	High	0.02	None
*C. melibiosica* 499/24	0.90	Weak	0.48	High	0.02	None

Hz index: high, Hz < 0.50; moderate, Hz = 0.50–0.69; weak, Hz = 0.70–0.99; none, Hz = 1.0. Pz index: high, Pz ≤ 0.63; weak, Pz = 0.64–0.99; none, Pz = 1.0. Biofilm formation (OD_595_): none (OD ≤ 0.131), weak (OD 0.132–0.262), moderate (OD 0.263–0.524), and high (OD > 0.524)

Among the *C. parapsilosis* isolates (*n* = 17), hemolysin production was notably variable. Three isolates showed no hemolytic activity (Hz = 1.00), ten demonstrated weak activity (Hz = 0.70–0.99), one was a moderate (Hz = 0.63), and three isolates exhibited strong hemolytic activity (Hz < 0.50) (Fig. 1A; Table 3).

### Phospholipase activity of *Candida* isolates

Pz values ranged from 0.36 to 1.00, reflecting substantial variation in enzymatic activity across the different species.

Isolates of *C. glabrata*, *C. krusei*, and *C. tropicalis* predominantly exhibited strong phospholipase activity (Pz ≤ 0.63). In contrast, *C. parapsilosis*, *C. albicans*, and *C. lusitaniae* showed Pz values spanning a wide numerical range (0.39 to 1.00). Notably, several *C. parapsilosis* isolates and one *C. krusei* isolate showed Pz values between 0.36 and 0.46—indicating the highest levels of phospholipase activity observed in this study (Fig. 1B; Table 3).

On the other hand, some isolates of *C. parapsilosis* were classified as non-producers (Pz = 1.00), indicating no detectable enzymatic activity (Fig. 1B; Table 3).

### Biofilm production by *Candida* isolates

The highest levels of biofilm production were observed in *C. parapsilosis*, *C. lusitaniae*, and *C. tropicalis*, with several strains exhibiting OD values exceeding 2.0—indicative of strong biofilm-forming ability. In contrast, *C. glabrata* showed no detectable biofilm formation (Fig. 1C; Table 3).

*Candida albicans* isolates showed varying biofilm OD values, with isolates distributed among non-producers, weak producers, and strong producers. Among the two *C. krusei* isolates, one was classified as a non-producer, while the other demonstrated strong biofilm production (Fig. 1C) (Table 3).

Based on OD_595_ values, biofilm formation was categorized into four levels: no production (OD ≤ 0.131), weak (OD 0.132–0.262), moderate (OD 0.263–0.524), and strong (OD > 0.524). According to this classification, approximately 77% of the isolates were classified as strong biofilm producers, underscoring the high prevalence of biofilm-forming capacity among the *Candida* isolates included in the study. Among the 23 neonates infected with isolates classified as strong biofilm producers, four (17.4%) died. Notably, these isolates belonged to *C. tropicalis*, *C. lusitaniae*, and *C. parapsilosis*—species consistently associated with elevated biofilm OD values among the clinical isolates. Additionally, in one of these fatal cases, the strain—identified as *C. lusitaniae*—also showed strong phospholipase activity. Detailed isolate-level results for hemolytic activity, phospholipase index, and biofilm formation are provided in the Supplementary Table 2.

## Discussion

The present study highlights the epidemiological and microbiological characteristics of neonatal candidemia in a tertiary care hospital in Brazil over a six-year period. The predominance of non-*albicans Candida* species observed aligns with recent global trends suggesting a shift from *C. albicans* to species such as *C. parapsilosis* and *C. tropicalis* [6, 26]. In our study, although *C. albicans* accounted for 22.8% of the isolates, non-*albicans* species collectively represented 77.2%, reflecting this changing epidemiological profile. This distribution may be influenced by factors such as antifungal selection pressure, enhanced biofilm-forming ability, and persistent environmental colonization [2, 27].

Recent epidemiological evidence also reinforces the shift toward non-*albicans Candida* species described in different clinical environments [28, 29]. Recent epidemiological study has shown a growing predominance of non-*albicans* species in invasive infections affecting both neonatal and adult populations. Consistent with this observation, a multicenter five-year surveillance study from Italy demonstrated that these species accounted for more than half of invasive candidiasis episodes and documented a progressive rise in antifungal resistance, particularly in *C. parapsilosis*, *C. glabrata*, and *C. tropicalis*. The same investigation reported that *C. lusitaniae* accounted for 14.7% of pediatric invasive candidiasis cases and that antifungal resistance among non-*albicans* species increased over the five-year period, with 69.12% of isolates resistant to azoles and 7.35% resistant to micafungin [28].

This study identified rare *Candida* species such as *C. melibiosica*, *C. pelliculosa*, and *C. pulcherrima*, emphasizing the need for precise species-level identification, especially in contexts with high antifungal pressure. Although infrequent, these emerging pathogens may pose therapeutic challenges. A pediatric study reported *C. pelliculosa* among bloodstream isolates with resistance to fluconazole and voriconazole [30], while a systematic analysis in China showed only 50% susceptibility of *C. pelliculosa* to fluconazole, indicating rising resistance in non-*albicans* species [31].

Broad-spectrum antibiotic use is a well-established risk factor for neonatal candidemia [14]. Prolonged antibiotic exposure has been associated not only with an increased risk of developing candidemia but also with cases of refractory infection [32]. The widespread use of antibacterial agents is believed to disrupt normal bacterial microbiota, facilitating *Candida* overgrowth and colonization [33]. Our findings are consistent with previous reports, including a multicenter study conducted across 11 NICUs in China, which similarly documented the routine use of broad-spectrum antibiotics for prophylactic or empirical treatment in neonatal populations [34].

Invasive procedures—central venous catheterization, mechanical ventilation, surgery, and parenteral nutrition—were common among neonates with candidemia [4, 35]. Parenteral nutrition alone has been previously implicated as a significant contributor to candidemia risk [36, 37]. Incorporating these into a risk stratification tool, like the *Candida* score, may aid in early intervention and reduce neonatal morbidity and mortality [38].

Although rare, polymicrobial candidemia was identified in two neonates, including one case with three different *Candida* species. Such occurrences may complicate treatment and indicate immunological vulnerability or extensive environmental colonization. A recent systematic review confirmed that multi-species fungemia, though uncommon, tends to occur in extremely premature neonates with low birth weight and central venous catheters, often requiring targeted antifungal therapy due to differing susceptibility profiles and being associated with higher mortality [39].

Persistent infections were observed in 10 out of 40 neonates (25.0%), which is in line with previous reports linking prolonged bloodstream infections to factors such as central venous catheter use, delayed initiation of antifungal therapy, or reduced antifungal susceptibility of the pathogen [14]. In this study, persistent candidemia was defined as continued positive blood cultures for ≥ 72 h after antifungal initiation, consistent with definitions used in multicenter studies on invasive fungal infections [17, 40]. The study reported a 45.0% mortality rate, aligning with previous national and international findings [2, 41]. Factors include delayed diagnosis, prematurity, and low birth weight. The average time to first positive *Candida* culture was 36.6 days, highlighting the need for early detection in high-risk neonates.

In this study, micafungin was commonly employed as a second-line antifungal, particularly when fluconazole proved ineffective or resistance was suspected. Modifications in antifungal therapy were required in 22.5% of cases, reflecting the complexity of treating neonatal *Candida* infections without prior susceptibility data. Although all isolates were susceptible, the observed variability in fluconazole MICs, particularly among non-*albicans* species, reflects the need for continuous monitoring of antifungal resistance, as previously highlighted by several studies correlating virulence profiles and antifungal responses [42]. Moreover, fluconazole exposure has been identified as a risk factor for *C. glabrata* infections [43], further emphasizing the importance of understanding local resistance patterns. These findings support the need for local surveillance and tailored antifungal strategies in NICUs [14, 44].

Virulence profiling demonstrated notable expression of pathogenic factors, including phospholipase and hemolysin activity and strong biofilm formation. Around two-thirds of the isolates, especially *C. parapsilosis*, *C. lusitaniae*, and *C. tropicalis*, were classified as strong biofilm producers. These traits are clinically significant due to their association with persistent infections on indwelling medical devices and reduced antifungal susceptibility. These findings are consistent with a previous study in which 74% of *Candida* isolates exhibited biofilm activity [45], and *C. tropicalis* was identified as the predominant biofilm-producing species in 47% of cases [46]. Our data similarly revealed high biofilm production in *C. tropicalis* isolates. Biofilm formation can adversely affect treatment outcomes by promoting catheter-related bloodstream infections and reducing therapeutic efficacy [8, 47, 48].

Interestingly, four neonates who died were infected with *Candida* strains classified as strong biofilm producers, and one of these isolates also exhibited high phospholipase activity, further supporting the potential cumulative effect of virulence factors on clinical outcomes. This interrelation between biofilm formation and phospholipase activity may contribute to an enhanced ability of *Candida* species to evade immune response and persist in the host, leading to more severe clinical outcomes [49, 50]. Although all tested isolates were categorized as susceptible according to established breakpoints, variability in fluconazole MICs—particularly among non-*albicans* species—may reflect differences in antifungal tolerance that could influence therapeutic response. This variability underscores the need for individualized treatment strategies based on both susceptibility profiles and virulence factor profiles.

Furthermore, persistent candidemia was observed in 25% of cases, and a considerable proportion of these required modification of antifungal therapy, illustrating the challenge of treating infections caused by virulent *Candida* strains with in vitro susceptibility, but persistent clinical infection. This is consistent with literature suggesting that virulence factors such as biofilm formation and enzyme production can contribute to treatment failure and clinical persistence despite in vitro susceptibility [51]. Previous studies have shown that biofilm formation and enzymatic activity are frequently detected among invasive *Candida* isolates and may contribute to persistence in the bloodstream or on indwelling devices [22, 52].

In addition to biofilm formation, phospholipase and hemolysin activity were frequently observed among the *Candida* isolates, enhancing their pathogenic potential. These enzymes aid in tissue invasion and immune evasion by degrading host membranes and modulating immune responses. A study in Uganda reported phospholipase activity in 94.3% and hemolysin production in 60% of *Candida* isolates, highlighting their role in virulence [22]. *Candida parapsilosis*, *C. lusitaniae*, and *C. tropicalis* showed particularly varied phospholipase profiles, with some strains exhibiting strong activity. Another study on neonatal bloodstream infections also detected phospholipase activity in both *C. albicans* and non-*albicans* species, reinforcing its relevance as a virulence factor in invasive neonatal *Candida* infections [49]. Although less prominent, hemolysin activity was detected in several isolates and may contribute to fungal survival by promoting erythrocyte lysis and iron acquisition [53]. The co-occurrence of multiple virulence factors underscores the need for integrated profiling to accurately assess pathogenicity and to better inform clinical decisions in the management of neonatal candidemia [9].

Despite its contributions, our study has several limitations. As a retrospective, single-center study with a relatively small sample size, the findings may not be generalizable to other NICU settings. Some clinical data were incomplete, particularly regarding follow-up and long-term outcomes. Additionally, fungal infections were only documented through culture-positive cases; non-culture-based diagnostics such as β-D-glucan (BDG) assays were not employed and may have led to underreporting of *Candida* infections [54, 55]. Moreover, the lack of molecular methods limited the depth of isolate characterization [56]. The statistical approach used in this study was intentionally aligned with its descriptive design and sample size. Simple univariable comparisons were selected because they appropriately reflect the structure of the dataset and allow meaningful clinical interpretation without overfitting or generating unstable estimates.

Among 23 neonates infected with strong biofilm-forming strains, four died—including one case involving *C. lusitaniae* with high phospholipase activity. These results suggest that strain-specific virulence factors, particularly biofilm formation and enzymatic activity, may be associated with increased disease severity and poorer clinical outcomes [52, 57]. This link between virulence traits and clinical severity has also been supported in recent studies. A meta-analysis involving 31 studies reported a mortality rate of approximately 70% in candidemia caused by biofilm-forming strains, nearly double the overall average of 37.9% [58]. When compounded by other virulence mechanisms such as phospholipase activity, as observed in one of our fatal cases, the pathogenic potential may be further amplified, supporting the clinical relevance of these findings [59, 60].

## Conclusion

In conclusion, the findings of this study underscore the need for integrated strategies to improve the prevention, diagnosis, and management of neonatal candidemia in NICUs. Given its characterization as a healthcare-associated infection, efforts should focus on targeted surveillance, antifungal stewardship, and routine microbiological profiling. Preventive measures—such as strict hand hygiene, implementation of infection control bundles, and monitoring of antimicrobial and nutritional practices—are essential to reduce incidence and improve patient safety. Future multicenter studies are warranted to validate these approaches and support the development of standardized protocols tailored to high-risk neonatal populations.

## Electronic Supplementary Material

Below is the link to the electronic supplementary material.

Supplementary file1 (XLSX 23.6 KB)

Supplementary file2 (XLSX 10.5 KB)

## Data Availability

The datasets generated and/or analyzed during the current study are available from the corresponding author upon reasonable request.
